# Natural Flavonoids Quercetin and Kaempferol Targeting G2/M Cell Cycle-Related Genes and Synergize with Smac Mimetic LCL-161 to Induce Necroptosis in Cholangiocarcinoma Cells

**DOI:** 10.3390/nu15143090

**Published:** 2023-07-10

**Authors:** Thanpisit Lomphithak, Patthorn Jaikla, Apiwit Sae-Fung, Sasiprapa Sonkaew, Siriporn Jitkaew

**Affiliations:** 1 Graduate Program in Clinical Biochemistry and Molecular Medicine, Department of Clinical Chemistry, Faculty of Allied Health Sciences, Chulalongkorn University, Bangkok 10330, Thailand; thanpisit.lomphithak@gmail.com (T.L.); sui.patthorn@gmail.com (P.J.); apiwit_fung@outlook.com (A.S.-F.); sasiprapa.sonk@gmail.com (S.S.); 2 Department of Clinical Chemistry, Faculty of Allied Health Sciences, Chulalongkorn University, Bangkok 10330, Thailand; 3 Center of Excellence for Cancer and Inflammation, Department of Clinical Chemistry, Faculty of Allied Health Sciences, Chulalongkorn University, Bangkok 10330, Thailand

**Keywords:** Cholangiocarcinoma, necroptosis, Smac mimetics, quercetin, kaempferol

## Abstract

Cholangiocarcinoma (CCA) is an aggressive cancer associated with a very poor prognosis and low survival rates, primarily due to late-stage diagnosis and low response rates to conventional chemotherapy. Therefore, there is an urgent need to identify effective therapeutic strategies that can improve patient outcomes. Flavonoids, such as quercetin and kaempferol, are naturally occurring compounds that have attracted significant attention for their potential in cancer therapy by targeting multiple genes. In this study, we employed network pharmacology and bioinformatic analysis to identify potential targets of quercetin and kaempferol. The results revealed that the target genes of these flavonoids were enriched in G2/M-related genes, and higher expression of G2/M signature genes was significantly associated with shorter survival in CCA patients. Furthermore, in vitro experiments using CCA cells demonstrated that quercetin or kaempferol induced cell-cycle arrest in the G2/M phase. Additionally, when combined with a Smac mimetic LCL-161, an IAP antagonist, quercetin or kaempferol synergistically induced RIPK1/RIPK3/MLKL-mediated necroptosis in CCA cells while sparing non-tumor cholangiocyte cells. These findings shed light on an innovative therapeutic combination of flavonoids, particularly quercetin and kaempferol, with Smac mimetics, suggesting great promise as a necroptosis-based approach for treating CCA and potentially other types of cancer.

## 1. Introduction

Cholangiocarcinoma (CCA) originates from cholangiocytes lining the biliary tree, both intrahepatic- and extrahepatic-bile ducts. Although the incidence of CCA in Thailand is almost 100 times higher than in other regions (approximately 85 per 100,000), the incidence rate has globally increased in recent decades [1]. CCA is highly heterogeneous and has an aggressive malignancy with a complexed tumor microenvironment [2,3]. Even though surgical resection represents the curative procedure in patients presenting in the early stages, the majority of patients are diagnosed with an unresectable disease in the advanced stages. Although there have been advancements in therapeutic strategies including targeted therapy and immunotherapy, chemotherapy remains the primary treatment for most CCA patients with initially unresectable diseases [2,4]. Currently, gemcitabine or gemcitabine combined with cisplatin is a standard regimen for first-line chemotherapy treatment of advanced CCA [5]. Nevertheless, the chemotherapy response rate is significantly low, leading to a poor prognosis [6,7]. Moreover, the 5-year survival rate of CCA patients in the advanced stages is relatively low, ranging from 5 to 10% [1]. Therefore, identifying novel therapeutic strategies to sensitize and improve chemotherapy response is urgently warranted.

We have previously reported that Smac mimetics sensitize gemcitabine to induce necroptosis in human CCA cells by degrading the cellular inhibitor of apoptosis proteins 1 and 2 (cIAP1/2) [8]. This strategy shows promise for CCA treatment by activating antitumor immunity through regulated cell death. Necroptosis involves the formation of a necrosome complex consisting of receptor-interacting protein kinase 1 and 3 (RIPK1 and RIPK3). RIPK3 then phosphorylates mixed lineage kinase domain-like (MLKL), leading to plasma membrane disruption and release of DAMPs (damage-associated molecular patterns) and tumor antigens [9,10,11,12]. Smac mimetics promote autoubiquitination and degradation of cIAP1/2, sensitizing cells to necroptosis [13,14,15,16]. Smac mimetics are being investigated in combination with chemotherapy for improved cancer treatment [17,18]. However, the use of gemcitabine and other DNA-damaging chemotherapeutic drugs with Smac mimetics may be limited due to side effects and drug resistance [19]. To address this, we explored natural compounds with anti-tumor activities that could be combined with Smac mimetics to induce necroptosis in CCA cells. These compounds should target cell cycle, DNA repair pathways, or inhibit proliferation, potentially offering multiple benefits by targeting various pathways involved in cancer cell growth.

Flavonoids are naturally occurring compounds found in vegetables and fruits. Quercetin and kaempferol are two noteworthy compounds among the flavonoids. Quercetin is abundantly found in foods such as onions, asparagus, and berries. On the other hand, kaempferol is primarily present in green leafy vegetables such as spinach and kale, as well as herbs such as dill, chives, and tarragon [20]. Flavonoids are considered as potential candidates for cancer prevention and treatment due to their safety, minimal side effects, high bioavailability, and cost-effectiveness [21,22,23,24]. According to the Structure-Activity Relationship (SAR), both quercetin and kaempferol belong to the flavonol subclass of flavonoids. They share a common basic structure consisting of two aromatic rings (A and B) connected by a three-carbon bridge (C ring). The main difference between quercetin and kaempferol lies in the positions of their hydroxyl groups. The SAR analysis of their structural features enables us to predict the activity of these compounds, including their potential anti-tumor activity [25,26]. Accumulating studies suggest that flavonoids, including quercetin and kaempferol, possess anti-tumor activities involving multiple molecular targets and mechanisms in various types of cancer, in both in vitro and in vivo models [23,27,28,29]. Several studies have reported that both quercetin and kaempferol cause cell cycle arrest and inhibit cell proliferation in various types of cancer [30,31,32,33,34]. Both quercetin and kaempferol have been shown to inhibit DNA topoisomerase II activity leading to DNA damage in HepG2 cells [35]. Kaempferol has been reported to induce DNA damage and inhibit DNA repair pathways [36]. In addition, both quercetin and kaempferol have been combined with chemotherapeutic drugs and radiotherapy to sensitize cancer cells and make them more sensitive to those treatments [37]. However, the combined anti-tumor activity of flavonoids, including quercetin and kaempferol, with Smac mimetics against human cancer cells, including CCA cells, remains to be elucidated.

Therefore, this current study aimed to comprehensively analyze the potential molecular targets of both quercetin and kaempferol related to cell-cycle regulation and DNA topoisomerase II using network pharmacology. Additionally, we investigated their associations with the prognosis of CCA patients using public databases and integrated bioinformatics tools. In vitro validation related to the effect of cell-cycle and proliferation inhibition was carried out in two representative CCA cells. Finally, we explored the novel therapeutic combination of quercetin or kaempferol with Smac mimetics to induce necroptosis in CCA cells.

## 2. Materials and Methods

### 2.1. Molecular Docking Analysis

#### 2.1.1. Protein Preparation

The crystallographic structure of DNA topoisomerase II (PDB ID: 5GWK) was obtained from the RCSB Protein Data Bank. To prepare the protein for docking, a previously established procedure was followed. In summary, all non-protein molecules were removed from the structure, and missing hydrogen atoms and Kollman charges were added using AutoDockTools-1.5.7 software (The Scripps Research Institute, La Jolla, CA, USA). The prepared protein structures were saved in a PDBQT format to be used in subsequent molecular docking studies.

#### 2.1.2. Ligand Preparation

The ligand structures, including etoposide (PubChem CID: 36462), quercetin (PubChem CID: 5280343), and kaempferol (PubChem CID: 5280863), were retrieved from the PubChem online database (https://pubchem.ncbi.nlm.nih.gov, accessed on 24 March 2023) in SDF format. The ligand structures were converted to a PDB format using BIOVIA Discovery Studio 2020 (Dassault Systèmes, San Diego, CA, USA) and then further converted to a PDBQT format using the AutoDockTools-1.5.7 program.

#### 2.1.3. Molecular Docking

Molecular docking studies were conducted using AutoDock Vina with default parameters to analyze the interactions between etoposide, quercetin, kaempferol, and topoisomerase II. The grid boxes were set with dimensions of 40 × 40 × 40 points, a spacing of 1 Å, and a center grid box positioned at coordinates 23.307 × −38.584 × −59.568 (xyz). The conformation with the lowest binding energy was selected, and the protein–ligand interaction was evaluated using BIOVIA Discovery Studio 2021 (Dassault Systèmes, San Diego, CA, USA).

### 2.2. Collection of Target Genes for Quercetin and Kaempferol

The potential target genes were collected from various sources, including SwissTargetPrediction (http://www.swisstargetprediction.ch/, accessed on 29 March 2023) [38], BindingDB (https://www.bindingdb.org/rwd/bind/index.jsp, accessed on 3 April 2023) [39], PharmMapper (http://lilab.ecust.edu.cn/pharmmapper/, accessed on 24 March 2023) [40], and Super-PRED (https://prediction.charite.de/, accessed on 29 March 2023) [41]. The SMILES format of Quercetin and Kaempferol was obtained from the PubChem database (https://pubchem.ncbi.nlm.nih.gov/, accessed on 24 March 2023) for use in SwissTargetPrediction and BindingDB. The structures of quercetin and kaempferol files (PubChem CID: 5280343 and 5280863) were uploaded into PharmMapper to predict possible targets. The names of quercetin and kaempferol were used to search for targets in Super-PRED. Additionally, a total of 157 cell cycle-related genes were obtained from the Kyoto Encyclopedia of Genes and Genomes (KEGG; https://www.kegg.jp/, accessed on 22 April 2023). Finally, a Venn diagram (http://bioinformatics.psb.ugent.be/webtools/Venn/, accessed on 2 May 2023) was used to identify the target genes shared by quercetin or kaempferol and cell cycle-related genes.

### 2.3. Protein-Protein Interaction (PPI) Network and Functional and Pathway Enrichment Analysis

A protein–protein interaction (PPI) network analysis was performed on the target genes using the STRING database (https://www.string-db.org/, accessed on 2 May 2023) [42]. In addition, to investigate the functional pathways that are more enriched in the set of the target genes in the PPI network than in the background, an enrichment analysis for Gene Ontology (GO), Reactome Pathway, and local network cluster (STRING) were obtained from the analysis section of the STRING database. The results are shown as a bubble chart, in which the false discovery rate (FDR) is represented by colors, the gene ratio is represented by bubble size, and the strength (enrichment effect) is represented in the X axis.

### 2.4. Overall Survival of Target Gene Expression in CCA Patients

The GSE76297 and GSE89749 datasets were downloaded from the Gene Expression Omnibus (GEO) database (https://www.ncbi.nlm.nih.gov/geo/, accessed on 8 May 2023). A comparative analysis of the 18 overlapping genes expressions was performed on 91 pairs of tumor and non-tumor tissues from the GSE76297 dataset using a paired-sample *t*-test presented in a box plot. A Kaplan–Meier and log-rank test were used to compare patient survival between G2/M groups using R statistical software (version 4.1.0) (The R Project for Statistical Computing, Indianapolis, IN, USA) with R’s “survminer” package. Survival data were obtained from the GSE89749 dataset. The G2/M signature group, which includes *CCNA2*, *CCNB1*, *CCNB2*, *CDC25B*, *CDC25C*, *CDK1*, *ABL1*, *AURKB*, *CHEK1*, and *PLK1*, was used to stratify patients into two groups. Each gene was initially divided into low and high groups based on the median as the cutoff, resulting in a score of 0 and 1, respectively. Subsequently, the scores of all genes in the G2/M signature were summed, and patients were stratified into low (scores ranging from 0 to 4) and high (scores ranging from 5 to 10) groups.

### 2.5. Reagents

LCL-161 was purchased from APExBIO Technology LLC (Houston, TX, USA). Pan-caspase inhibitor, zVAD-fmk (carbobenzoxy-valyl-alanyl-aspartyl-[O-methyl]- fluoromethylketone), GSK’782, and necrosulfonamide (NSA) were purchased from Calbiochem (Merck Millipore, Darmstadt, Germany). Quercetin, kaempferol, and necrostatin-1 were purchased from Sigma-Aldrich (St. Louis, MO, USA) and dissolved in dimethyl sulfoxide (DMSO) following the manufacturer’s instructions to prepare stock solutions. Working solutions were subsequently prepared by diluting the stock solutions and ensuring that they contained a non-toxic concentration of DMSO, less than 0.15%.

### 2.6. Cell Lines and Culture

The HuCCT-1 cell line, derived from human Cholangiocarcinoma (CCA), was obtained from the Japanese Collection of Research Bioresources (JCRB) Cell Bank in Osaka, Japan. The RMCCA-1 cell line, also a human CCA cell line, was developed from Thai CCA patients [43]. Both HuCCT-1 and RMCCA-1 cells were cultured in HAM’s F-12 medium (HyClone Laboratories, Logan, UT, USA) supplemented with 10% fetal bovine serum (Sigma, St Louis, MO, USA) and 1% Penicillin-Streptomycin (HyClone Laboratories, Logan, UT, USA). All cell lines were maintained in a humidified incubator at 37 °C with 5% CO_2_. Furthermore, rigorous testing confirmed that there was no mycoplasma contamination in any of the cell lines.

### 2.7. Cell Cycle Analysis

CCA cells were treated with quercetin or kaempferol as specified concentrations for 48 h. Following the treatment, cell-cycle analysis was conducted by employing propidium iodide (PI) staining to assess DNA content. Briefly, the cells were fixed with 70% ethanol and subsequently washed with Phosphate Buffered Saline (PBS; HyClone Laboratories, Logan, UT, USA). The cells were then resuspended in PBS containing 0.25% Triton X, along with RNase A (100 μg/mL) and PI (50 μg/mL) and incubated for 30 min. Finally, the stained cells were analyzed using flow cytometry for further examination. Data analysis was then performed using the FlowJo™ version 10 software (Becton Dickinson and Company (BD) Franklin Lakes, NJ, USA).

### 2.8. Cell Death Induction and Detection by Annexin V/PI Staining

For the treatment conditions involving Smac mimetic LCL-161 (5 μM for RMCCA-1 and 25 μM for HuCCT-1) and zVAD-fmk (20 μM), the inhibitors were pre-treated for a minimum of 2 h followed by treatment with quercetin or kaempferol (concentrations as indicated). Cell death was assessed through Annexin V-FITC and PI staining, followed by flow cytometry analysis. In brief, cells were washed and then suspended in Annexin V binding buffer containing recombinant Annexin V-FITC (ImmunoTools, Friesoythe, Germany) and PI (Invitrogen, Carlsbad, CA, USA). The stained cells were examined using a flow cytometer (Navios, Beckman Coulter, Indianapolis, IN, USA). A total of ten thousand events were collected for each sample, and the data were analyzed using Navios software. The combination index (CI) was calculated based on the Chou-Talalay method, where CI values of 1, <1, and >1 indicate an additive effect, synergism, and antagonism, respectively [44].

### 2.9. Lentivirus Infection of CRISPR and shRNA Constructs

Lentiviral plasmids carrying shRNA constructs were obtained from Sigma (St. Louis, MO, USA). The shRNA sequences targeted human MLKL (NM_152649.4) at the 3′ untranslated regions 2025–2045 (shMLKL#1) and 1907–1927 (shMLKL#2). For CRISPR experiments, plasmids targeting human RIPK1 (NM_003804) and RIPK3 (NM_006871) were generated following Zhang’s protocol [45]. The sequences for CRISPR-RIPK1 and CRISPR-RIPK3 were 5′-CACCGGATGCACGTGCTGAAAGCCG-3′ and 5′-CAGTGTTC-CGGGCGCAACAT-3′, respectively. All plasmid constructs were validated by DNA sequencing. To generate lentiviral particles, HEK293T cells were co-transfected with packaging plasmid (pCMV-VSV-G) and envelope plasmid (pCMV-dr8.2-dvpr), along with either shRNA-non-targeting (shNT; pLKO.1puro), shRNA-MLKL (shMLKL#1 or shMLKL#2), CRISPR-V2, CRISPR-RIPK1, or CRISPR-RIPK3 plasmids. After 24 h, supernatants were collected and passed through a 0.22 μm sterile filter membrane (Jet Bio-Filtration, Guangzhou, China) to obtain viral particles. The lentiviral preparation was then used to infect CCA cells in the presence of 8 μg/mL polybrene (Merck Millipore, Darmstadt, Germany). After 24 h of infection, cells were further cultured in the presence of puromycin (Merck Millipore, Darmstadt, Germany) for 48 h to select for successfully transduced cells.

### 2.10. Western Blot Analysis

The cells were washed twice with ice-cold PBS and lysed in RIPA buffer (Merck Millipore, Darmstadt, Germany) supplemented with a proteinase inhibitor cocktail (Roche, Mannheim, Germany) on ice for 30 min. The total protein concentrations were determined using the Bradford assay (Bio-Rad, Hercules, CA, USA). Total proteins (20–50 μg) were separated by 10–20% SDS-PAGE and transferred onto PVDF membranes. The membranes were blocked with a 5% blotting-grade blocker (Bio-Rad, Hercules, CA, USA) at room temperature for 1 h, followed by overnight incubation with primary antibodies at 4 °C. The primary antibodies used in this study were anti-RIPK1 (610459) from BD Biosciences (San Jose, CA, USA), anti-MLKL (ab184718) and anti-phosphorylated MLKL (ab187091) from Abcam (Cambridge, UK), and anti-RIPK3 (8457) and anti-β-Actin (4970) from Cell Signaling (Danvers, MA, USA). After incubation with primary antibodies, the blots were washed three times with TBS-T (Tris-buffered saline, 0.5% Tween 20) buffer and incubated with horseradish peroxidase-conjugated secondary antibodies (Cell Signaling Technology, Danvers, MA, USA) at room temperature for 1 h. The proteins were visualized using enhanced chemiluminescence according to the manufacturer’s instructions (Bio-Rad, Hercules, CA, USA) using Amersham ImageQuant 800 Western blot imaging systems. All the Western blots shown are representative of at least three independent experiments.

### 2.11. Statistical Analysis

All data were analyzed using either R statistical software (version 4.1.0) or SPSS (version 22.0, IBM Corp; Armonk, NY, USA). Bioinformatics analysis involved the use of Wilcoxon or Student’s *t*-tests to assess group differences. The log-rank test was utilized to examine variations in survival between G2/M signature groups, with Kaplan-Meier curves used to visualize patient survival. Pearson’s correlation coefficient (r) was employed for all correlation analyses. For in vitro experiments, the results were presented as the mean ± standard deviation (S.D.) of at least three independent experiments. Student’s *t*-test was conducted to compare two groups. Statistical significance was defined as a *p*-value < 0.05, indicated by asterisks (* *p* < 0.05, ** *p* < 0.01, *** *p* < 0.001, **** *p* < 0.0001).

## 3. Results

### 3.1. Molecular Docking of Quercetin or Kaempferol with DNA Topoisomerase II

**Figure 1:**
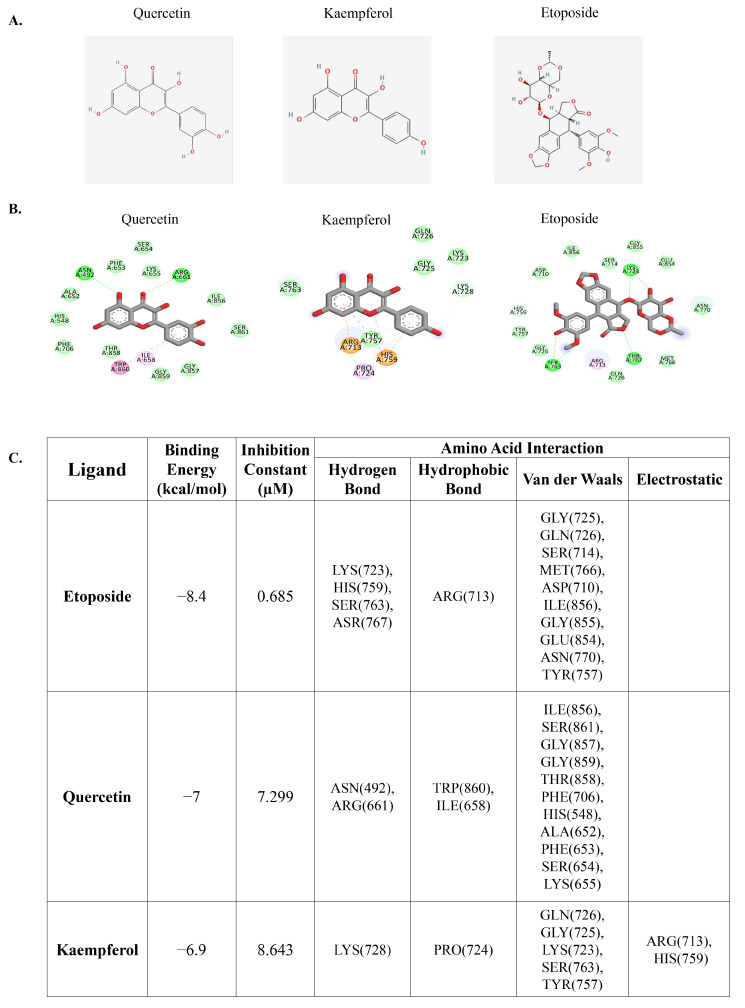
Molecular docking of quercetin, kaempferol, and DNA topoisomerase II. (**A**) 2D chemical structure depiction of quercetin, kaempferol, and etoposide. (**B**) Schematic interactions of amino acid residues between quercetin or kaempferol or etoposide as a reference compound and DNA topoisomerase II. The green dashed line represents hydrogen bonds, the pink or purple dashed lines represent hydrophobic bonds, and the yellow dashed line indicates other bonds. (**C**) Molecular docking results indicating binding energy and amino acid interactions.

Previous research has demonstrated that flavonoids, including quercetin and kaempferol, with multiple phenolic rings can interact and inhibit DNA topoisomerase II similar to the chemotherapeutic drug etoposide, which can induce DNA double-strand breaks [35]. The induction of such DNA damage is crucial for its anti-cancer effects, as it disrupts the process of DNA replication and leads to cell-cycle arrest and eventual cell death. We initially investigated the potential binding interactions between quercetin, kaempferol, and DNA topoisomerase II using molecular docking analysis. Molecular docking analysis is a valuable tool in drug discovery and development, enabling researchers to predict and evaluate the binding interactions between potential therapeutic agents and target proteins. The results demonstrated that etoposide, a well-known molecule that targets DNA topoisomerase II, as well as the native molecules from the complex used in the analysis, had a binding energy of −8.4 kcal/mol, while quercetin and kaempferol had binding energies of −7 kcal/mol and −6.9 kcal/mol, respectively (Figure 1A–C). These results suggest that quercetin and kaempferol have the potential to bind to DNA topoisomerase II.

### 3.2. Identifying Cell Cycle-Related Gene Targets of Quercetin or Kaempferol

**Figure 2:**
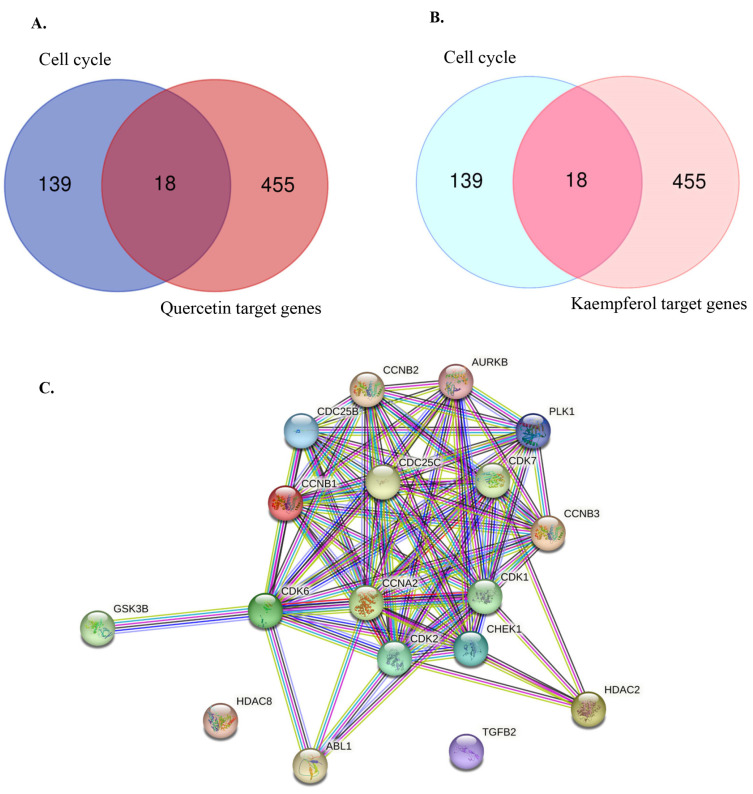
Identification of overlapping genes between quercetin, kaempferol target genes, and cell cycle-related genes. (**A**) Venn diagram representing the overlapping of cell cycle-related gene targets (157 genes) and quercetin targets (473 genes). (**B**) Venn diagram representing the overlapping of cell cycle-related gene targets (157 genes) and kaempferol targets (473 genes). (**C**) Protein–protein interaction (PPI) analysis of 18 overlapping genes.

**Figure 3:**
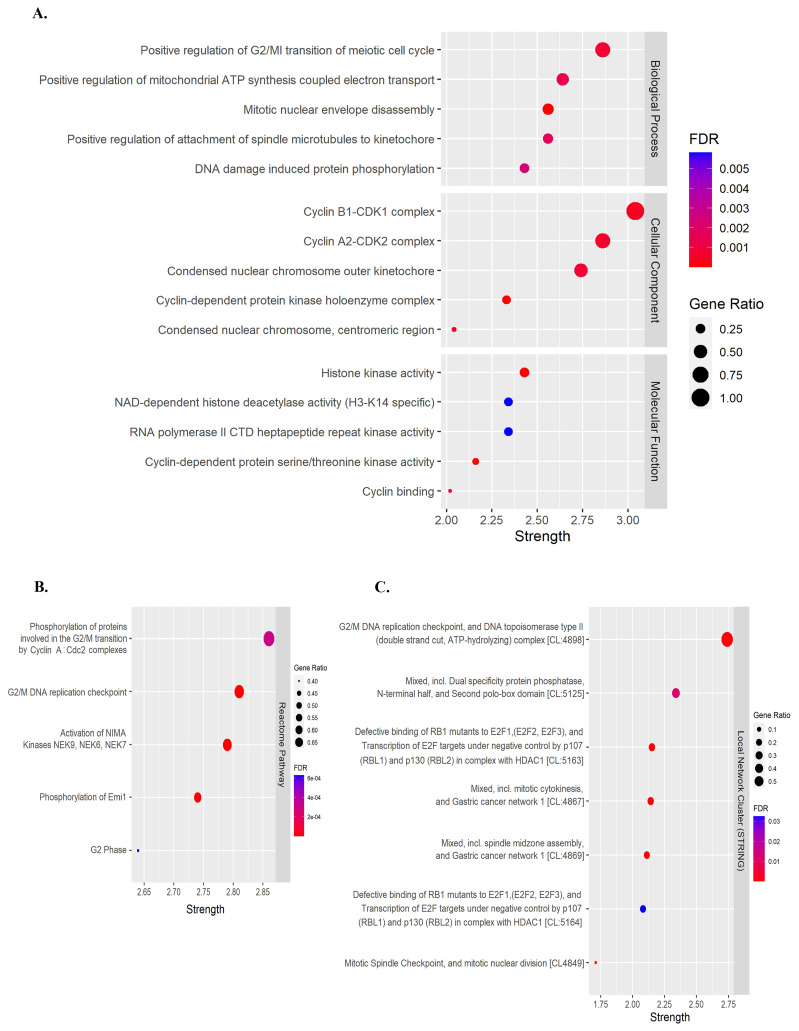
Functional pathway enrichment analysis of overlapping target genes. (**A**) Gene Ontology (GO) pathway enrichment analysis. (**B**) Reactome pathway enrichment analysis. (**C**) Local network cluster (STRING) enrichment analysis.

Previous studies have demonstrated that flavonoids, including quercetin and kaempferol, cause cell-cycle arrest and inhibit cell proliferation in various types of cancer [30,31,32,33,34]. Therefore, to identify the target genes of quercetin and kaempferol related to cell-cycle regulation, the targets of quercetin and kaempferol were predicted from databases including SwissTargetPrediction, BindingDB, PharmMapper, and Super-PRED. After merging the targets for both compounds, a total of 473 targets were obtained. From these targets, 18 genes were selected based on their intersection with cell cycle-related genes (*ABL1*, *AURKB*, *CCNA2*, *CCNB1*, *CCNB2*, *CCNB3*, *CDC25B*, *CDC25C*, *CDK1*, *CDK2*, *CDK6*, *CDK7*, *CHEK1*, *GSK3B*, *HDAC2*, *HDAC8*, *PLK1*, and *TGFB2*) (Figure 2A,B). To study the relationship between the selected target genes, a PPI network and functional enrichment analyses were performed using the STRING database. The PPI network demonstrated associations between 18 target genes (Figure 2C). In the Gene Ontology enrichment analyses, positive regulation of the G2/M transition of the meiotic cell cycle was significantly enriched in the Biological Process (Figure 3A). Phosphorylation of proteins involved in the G2/M transition by Cyclin A:Cdc2 complexes and G2/M DNA replication checkpoint were significantly enriched in the reactome pathway (Figure 3B). Moreover, in the local network cluster (STRING), G2/M DNA replication checkpoint, and DNA topoisomerase type II (double strand cut, ATP-hydrolyzing) complex was significantly enriched (Figure 3C). These results suggest that both quercetin and kaempferol might target cell cycle-related genes, particularly the G2/M cell-cycle phase.

### 3.3. G2/M Cell Cycle Target Genes of Quercetin or Kaempferol Are Associated with Shorter Survival in CCA Patients

**Figure 4:**
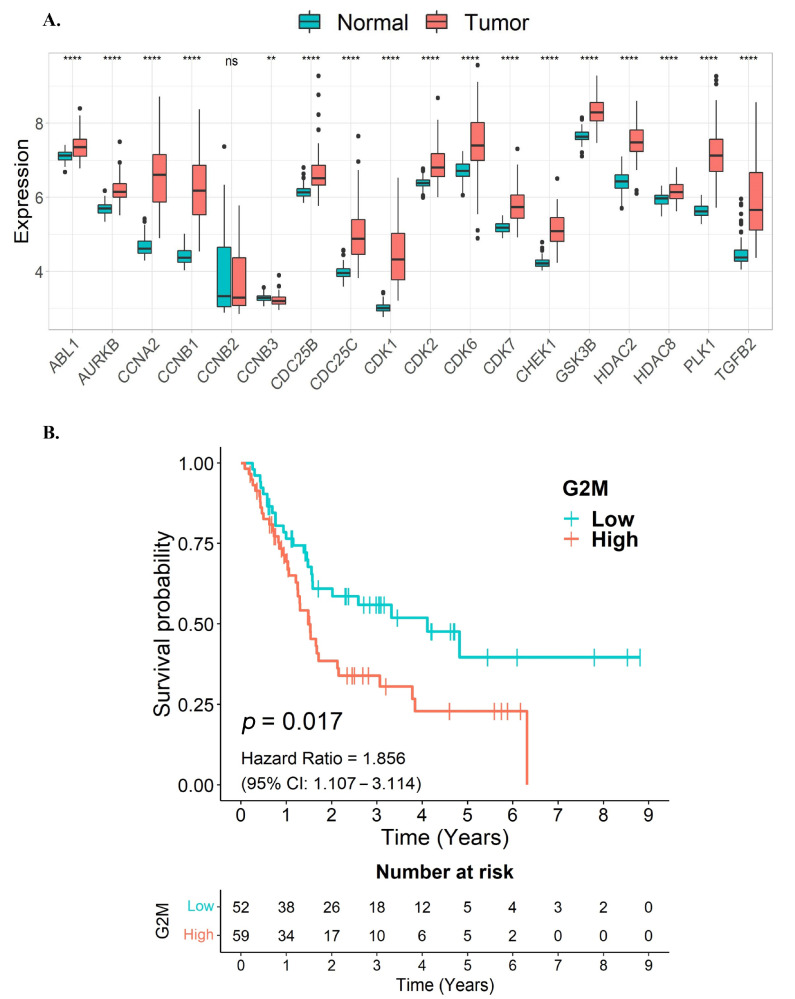
Association of G2/M cell cycle-related target genes of quercetin or kaempferol with overall survival in CCA patients. (**A**) The expression levels of quercetin and kaempferol target genes associated with cell-cycle regulation in tumor tissues compared to non-tumor adjacent tissues presented as a box plot. (**B**) A Kaplan–Meier curve showing the overall survival of CCA patients between high and low expression levels of the G2/M signature gene set. ns, not significant; ** *p* < 0.01; **** *p* < 0.0001.

To examine the relationship between the expression levels of quercetin and kaempferol target genes associated with cell-cycle regulation and overall survival in CCA samples, we utilized data from the GSE76297 and GSE89749 cohorts. Initially, we assessed the expression levels of these target genes in the CCA samples. Notably, our findings indicated that the majority of the quercetin and kaempferol target genes associated with cell-cycle regulation, out of the 18 overlapping genes, were significantly upregulated in tumor tissues compared to non-tumor adjacent tissues (Figure 4A). As these 18 overlapping genes were predominantly enriched in G2/M cell-cycle associated pathways, we further constructed a G2/M gene signature by including six genes identified in our analysis (*CCNA2*, *CCNB1*, *CCNB2*, *CDC25B*, *CDC25C*, and *CDK1*), in addition to four other G2/M hallmark genes from the MSigDB database (*ABL1*, *AURKB*, *CHEK1*, and *PLK1*). Our results revealed a significant association between high expression levels of the G2/M signature gene set and poorer survival in CCA patients (*p* = 0.017, HR = 1.856) (Figure 4B). These results suggest that the elevated expression of the G2/M gene signature, associated with unfavorable survival outcomes in CCA patients, might be potential targets for CCA therapy.

### 3.4. Quercetin or Kaempferol Cause G2/M Cell Cycle Arrest in CCA Cells

**Figure 5:**
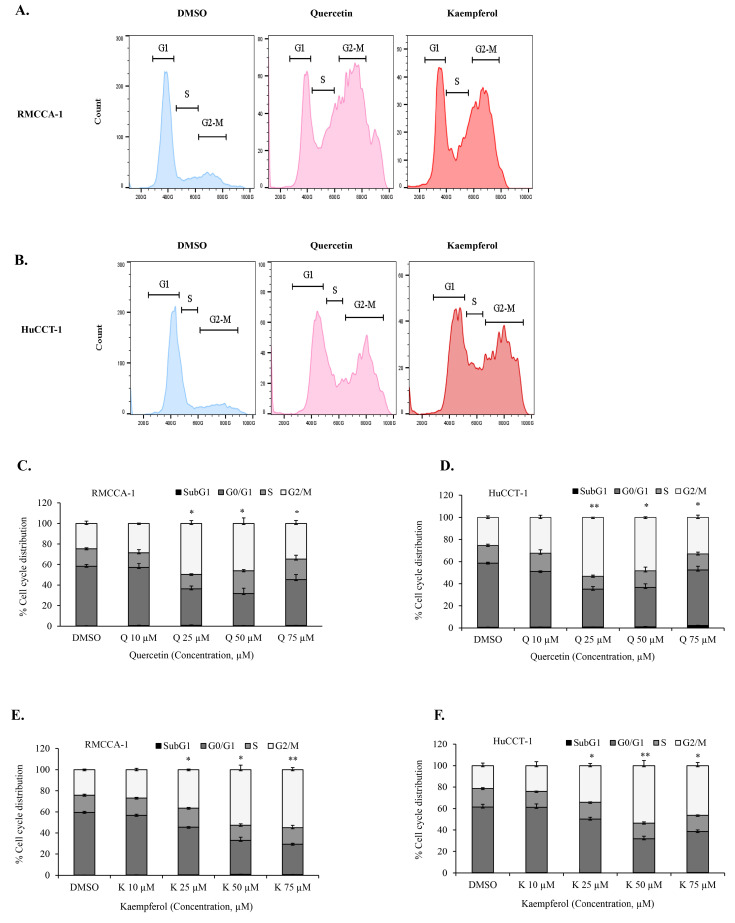
Induction of G2/M cell cycle arrest in CCA cells by quercetin or kaempferol. Representative histograms demonstrating cell-cycle analysis in RMCCA-1 (**A**) and HuCCT-1 (**B**) treated with quercetin or kaempferol. Cell-cycle analysis by flow cytometry using propidium iodide staining of RMCCA-1 (**C**) and HuCCT-1 (**D**) treated with indicated concentrations of quercetin and kaempferol in RMCCA-1 (**E**) and HuCCT-1 (**F**), respectively. * *p* < 0.05, ** *p* < 0.01.

Our bioinformatics analysis identified genes in G2/M cell-cycle associated pathways as targets of quercetin and kaempferol. To further investigate whether quercetin and kaempferol can induce G2/M cell-cycle arrest, we conducted in vitro experiments using CCA cell models, RMCCA-1 and HuCCT-1. The effects of quercetin and kaempferol on the cell cycle in CCA cells were examined. We analyzed the cell-cycle perturbations induced by quercetin and kaempferol, as depicted in the representative histograms (Figure 5A,B). The results revealed that treatment with different concentrations of quercetin (Figure 5C,D) or kaempferol (Figure 5E,F) resulted in a significant arrest of CCA cells in the G2/M phase for both the RMCCA-1 and HuCCT-1 cell lines. These findings demonstrated the ability of quercetin and kaempferol to effectively induce G2/M cell-cycle arrest in CCA cells.

### 3.5. Quercetin or Kaempferol Synergize with Smac Mimetics to Induce Cell Death in CCA Cells

**Figure 6:**
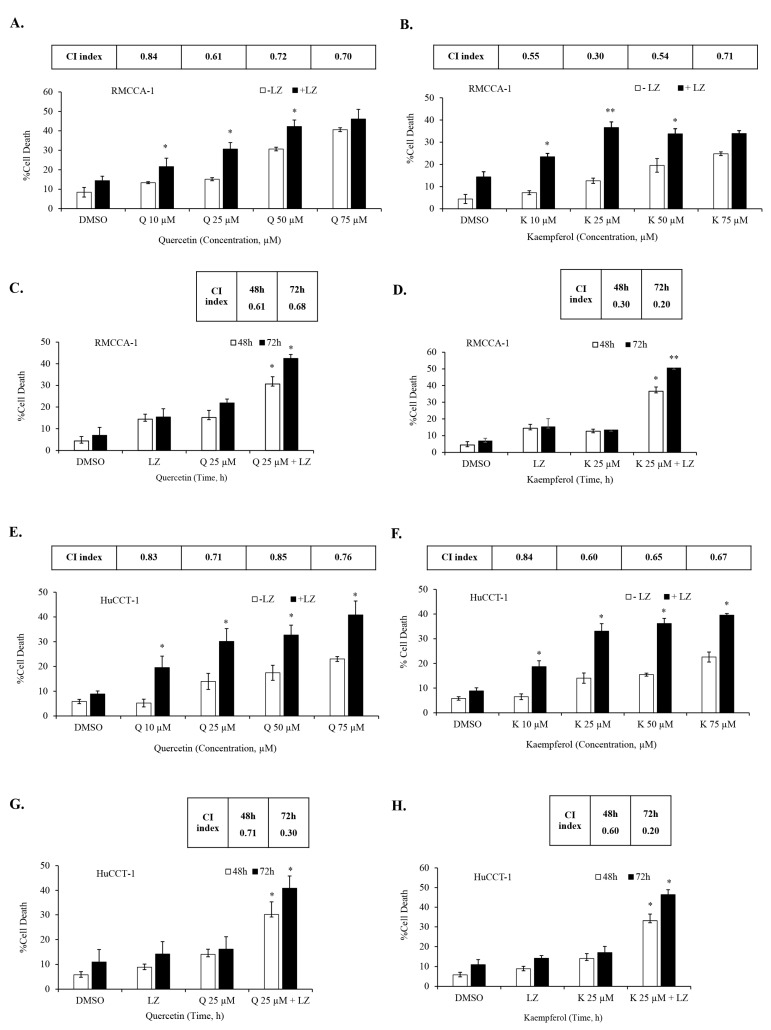
Synergistic induction of cell death in CCA cells by quercetin or kaempferol in combination with Smac mimetic. RMCCA-1 was treated with indicated concentrations of quercetin (**A**) and kaempferol (**B**) in the presence or absence of Smac mimetic LCL-161 and pan-caspase inhibitor (zVAD-fmk). Combination treatment of quercetin (**C**) or kaempferol (**D**) with Smac mimetic LCL-161 in RMCCA-1 at 48 h and 72 h. HuCCT-1 was treated with indicated concentrations of quercetin (**E**) and kaempferol (**F**). Combination treatment of quercetin (**G**) or kaempferol (**H**) with Smac mimetic LCL-161 in HuCCT-1 at 48 h and 72 h. Cell death was determined by Annexin V and PI staining and flow cytometry. Combination index (CI) was calculated. Data presented as mean ± S.D. of three independent experiments are shown; * *p* < 0.05, ** *p* < 0.01, LZ; LCL-161 + zVAD-fmk.

**Figure 7:**
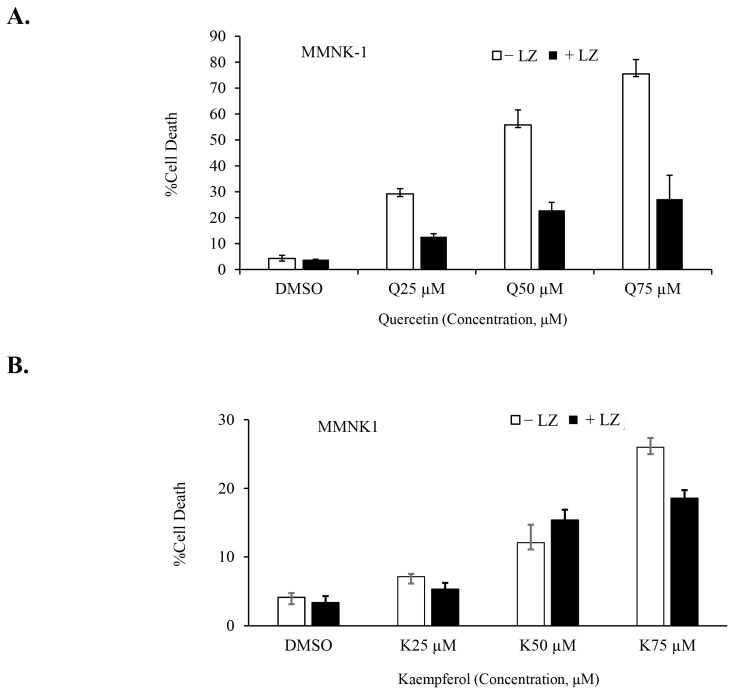
No cell death induction in a non-tumor cholangiocyte treated with the combination of quercetin or kaempferol and Smac mimetic. MMNK-1, a non-tumor cholangiocyte cell line, was treated with indicated concentrations of quercetin (**A**) and kaempferol (**B**) in the presence or absence of Smac mimetic LCL-161 and pan-caspase inhibitor (zVAD-fmk). Cell death was determined by Annexin V and PI staining and flow cytometry. Data presented as mean ± S.D. of three independent experiments are shown; LZ; LCL-161 + zVAD-fmk.

In our previous studies, we demonstrated the potential of Smac mimetics to synergize with different inducers and induce necroptosis in CCA cells [46,47], particularly in combination with the chemotherapeutic agent gemcitabine [8]. In this study, we aimed to investigate whether the Smac mimetic LCL-161 (currently under clinical trial evaluation) could synergize with quercetin or kaempferol to induce cell death in CCA cells. To evaluate this, we treated CCA cells to various concentrations of quercetin (Figure 6A) and kaempferol (Figure 6B) with and without LCL-161, in the presence of the pan-caspase inhibitors; zVAD-fmk. The results demonstrated a significant synergistic induction of cell death in both RMCCA-1 and HuCCT-1 CCA cells by quercetin and kaempferol when combined with Smac mimetic LCL-161, as indicated by the combination index (CI index) values below 1. To determine the highest synergistic combination, we selected the treatment with the lowest CI index and applied it to treat CCA cells for 48 and 72 h. Subsequently, we observed a time-dependent increase in CCA cell death upon treatment with the combination of quercetin or kaempferol with Smac mimetic LCL-161 (Figure 6C,D,G,H). To provide a normal comparative control, we also investigated the combination treatment in MMNK-1, a non-tumor cholangiocyte cell line. Interestingly, the combination of either quercetin or kaempferol with Smac mimetic LCL-161, in the presence of pan-caspase inhibitors, did not induce cell death in the MMNK-1 cell line (Figure 7A,B). Overall, our findings indicate a synergistic effect between quercetin or kaempferol and Smac mimetic LCL-161 in inducing cell death specifically in CCA cells. This highlights the potential therapeutic value of combining these agents for targeted treatment of CCA patients.

### 3.6. Combination of Quercetin or Keampferol and Smac Mimetic Induce Cell Death through Necroptosis in CCA Cells

**Figure 8:**
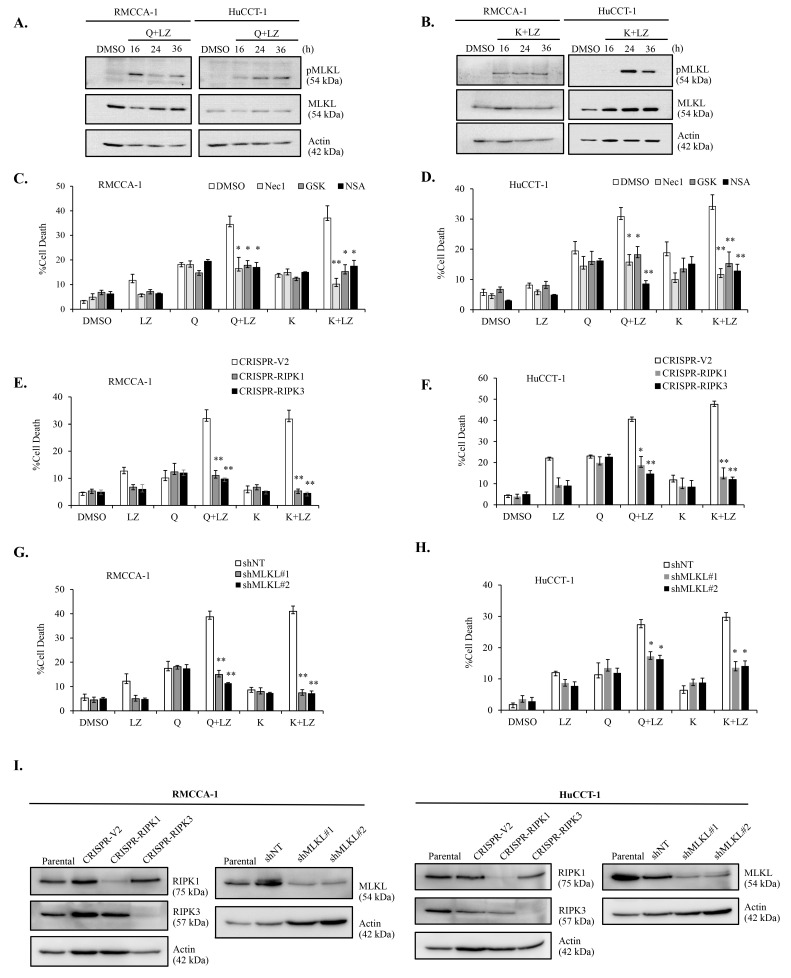
Induction of cell death through necroptosis in CCA cells by the combination of quercetin or kaempferol and Smac mimetics. Western blot analysis demonstrating pMLKL, a specific marker of necroptosis activation, in RMCCA-1 (**A**) and HuCCT-1 (**B**) treated with quercetin or kaempferol in combination with Smac mimetic LCL-161 and pan-caspase inhibitor (zVAD-fmk). Inhibition of cell death induced by the combination treatment of quercetin or kaempferol and Smac mimetics in RMCCA-1 (**C**) and HuCCT-1 (**D**) cells using the RIPK1 inhibitor (30 μM Nec-1), RIPK3 inhibitor (10 μM GSK’872), and the MLKL inhibitor (1 μM NSA). Reduced cell death in RIPK1 or RIPK3 knockout in RMCCA-1 (**E**) and HuCCT-1 (**F**) treated with the combination of quercetin or kaempferol and Smac mimetic. Reduced cell death in MLKL knockdown in RMCCA-1 (**G**) and HuCCT-1 (**H**) treated with the combination of quercetin or kaempferol and Smac mimetics. Cell death was determined by Annexin V and PI staining and flow cytometry (**I**) Confirmation of the successful depletion of RIPK1, RIPK3, and MLKL proteins in CCA cells by Western blot analysis. Data presented as mean ± S.D. of three independent experiments are shown: * *p* < 0.05, ** *p* < 0.01. LZ; LCL-161 + zVAD-fmk.

To validate necroptosis as the form of cell death induced by the combination of quercetin or kaempferol with Smac mimetic LCL-161, we employed multiple approaches to confirm necroptosis. Initially, we investigated the activation of phosphorylated MLKL (pMLKL), a specific marker of necroptosis, using Western blot analysis. The results demonstrated that the combination treatment of quercetin (Figure 8A) or kaempferol (Figure 8B) with Smac mimetic LCL-161 led to an increase in pMLKL expression in both RMCCA-1 and HuCCT-1 cells. Furthermore, we employed pharmacological inhibitors targeting key necroptotic proteins, including necrostatin-1 (Nec-1), GSK’872 (GSK), and necrosulfonamide (NSA), which are specific inhibitors of RIPK1, RIPK3, and MLKL, respectively. The results revealed that all inhibitors significantly inhibited cell death induced by the combination treatment of quercetin or kaempferol with Smac mimetic LCL-161 in both RMCCA-1 and HuCCT-1 cells (Figure 8C,D). Moreover, we generated CCA cells lacking key necroptotic proteins using genetic inhibition. CRISPR/Cas9 technology was employed to generate RIPK1 and RIPK3 knockout CCA cells, while shRNA was used to achieve MLKL knockdown in CCA cells. The successful depletion of these proteins was confirmed (Figure 8I). Our findings demonstrated that the loss of *RIPK1* or *RIPK3* in CCA cells significantly suppressed cell death induced by the combination treatment (Figure 8E,F). Similarly, the absence of *MLKL* provided protection to CCA cells against cell death induction by the combination treatment of quercetin or kaempferol with Smac mimetic LCL-161 (Figure 8G,H). Collectively, our comprehensive investigations utilizing various approaches consistently confirm that the combination of quercetin or kaempferol with Smac mimetic LCL-161 when caspases were inhibited, induces cell death in CCA cells through RIPK1/RIPK3/MLKL-mediated necroptosis.

## 4. Discussion

Several studies have demonstrated the improvement of Smac mimetics as a monotherapy treatment by exploring the rationale combination of cytotoxic drugs and DNA-damaging agents in the context of chemotherapy with Smac mimetics. This study is the first to explore the novel potential combination of flavonoids, specifically quercetin and kaempferol, with Smac mimetics. Several key findings were identified in the present study. First, we identified the molecular targets related to G2/M cell-cycle regulators of quercetin and kaempferol. These target genes are associated with shorter overall survival of CCA patients, suggesting that G2/M cell-cycle regulators could be potential therapeutic targets for CCA therapy. Second, using an in silico molecular docking approach, we observed potential interactions between quercetin and kaempferol and DNA topoisomerase II. Third, we have shown that both quercetin and kaempferol can effectively arrest CCA cells in the G2/M cell-cycle phase. This effect is likely due to the ability of quercetin and kaempferol to target multiple G2/M cell-cycle regulators and DNA topoisomerase II. Fourth, we have demonstrated that the Smac mimetic LCL-161 sensitizes CCA cells to necroptosis when caspases are inhibited in an RIPK1/RIPK3/MLKL-dependent manner. This effect was demonstrated through experiments utilizing both pharmacological inhibitors and genetic approaches. Notably, the combination effect was observed at relatively lower cytotoxic doses of quercetin and kaempferol. Lastly, the synergistic combination of flavonoids (quercetin and kaempferol) and Smac mimetics specifically induced necroptosis in CCA cell lines, while the non-tumor cholangiocyte remained resistant. Our findings provide a novel therapeutic combination between flavonoids, specifically quercetin and kaempferol, with Smac mimetics. This innovative combination holds potential for the development of a necroptosis-based therapeutic approach, not only for CCA but also for other cancer types.

Necroptosis can be considered as immunogenic cell death (ICD) due to its dual beneficial roles in killing tumor cells and inducing antitumor immunity [48]. Studies conducted in both in vitro and in vivo models across various types of cancer have shown that necroptosis represents a promising target for effective cancer treatments and opens up opportunities for overcoming apoptosis resistance [49,50,51,52,53,54,55]. Moreover, the combination of necroptosis activation and immune checkpoints inhibitors (ICIs) targeting the PD-1/PD-L1 pathway has been shown to improve treatment efficacy and survival in mouse models of melanoma and colorectal cancer compared to monotherapy [54]. Since the majority of CCA patients are initially diagnosed at advanced stages, and the tumor microenvironment of CCA is characterized by desmoplasia and immunosuppression [2,3], current therapeutic approaches have proven ineffective. Therefore, targeting necroptosis has the potential to be a promising therapeutic strategy for CCA patients. We have previously demonstrated that the activation of necroptosis, as indicated by the analysis of the specific marker phosphorylated MLKL (pMLKL) in human CCA tissues, is associated with a high infiltration of CD8+ T cells. This suggests that targeting necroptosis could be a promising therapeutic approach for CCA patients [47]. To further validate if necroptosis can be triggered in vitro in CCA cell models, we utilized two promising necroptosis inducers, gemcitabine or poly(I:C), a toll-like receptor 3 (TLR3) agonist, in combination with the Smac mimetic SM-164. Interestingly, the combination of both gemcitabine or poly(I:C) and the Smac mimetic SM-164 synergistically enhanced the induction of necroptosis in CCA cells expressing RIPK1/RIPK3/MLKL when caspases were inhibited [8,46]. Since poly(I:C) exhibited lower sensitization of necroptotic cell death than gemcitabine in CCA cells, gemcitabine becomes a more promising combination agent. However, due to the adverse side effect of gemcitabine and the development of drug resistance, in this current study, we aimed to rationally identify potential combination agents with Smac mimetics that have minimal side effects and target multiple genes.

Natural compounds with functional properties similar to chemotherapeutic drugs, such as DNA-damaging effects, targeting cell-cycle or DNA repair pathways, or inhibiting cell proliferation are promising candidates. Initially, we identified these natural compounds through a literature search. Among them, natural flavonoids, particularly quercetin and kaempferol, have been extensively studied and gained importance as cytotoxic and cytostatic agents, exhibiting the aforementioned functional properties. However, the combination of flavonoids (quercetin and kaempferol) with Smac mimetics has not yet been evaluated both in CCA or other cancers. To identify potential targets related to the cell cycle regulators of quercetin and kaempferol, we collected a list of potential target genes of quercetin and kaempferol and overlapped them with a set of 157 cell cycle-related genes. A total of 18 overlapping genes were identified. Furthermore, the protein–protein interaction (PPI) network and functional pathway enrichment analysis of these 18 overlapping target genes revealed that most of the overlapping target genes were enriched in the G2/M cell cycle-associated pathways. Specifically, the following G2/M cell cycle-related genes including *CDC25C* and *CDC25B*, were enriched in the Gene Ontology (GO) pathway enrichment analysis; *CDK1*, *CCNA2*, *CCNB1*, and *CCNB2* were enriched in the reactome pathway enrichment analysis; and finally, *CDK1*, *CCNB1*, and *CCNB2* were enriched in the local network cluster (STRING) enrichment analysis. This study is the first to utilize network pharmacology to identify potential target genes of flavonoids (quercetin and kaempferol) in the G2/M cell cycle-related pathways. Although, we did not validate the expression changes of these target genes following quercetin and kaempferol treatment, in vitro functional experiments confirmed that both quercetin and kaempferol dramatically arrested CCA cells in the G2/M cell-cycle phase. This effect may be attributed to their targeting of multiple genes related to the G2/M cell-cycle phase. Our findings are consistent with previous studies that have demonstrated the ability of both quercetin and kaempferol to induce G2/M cell-cycle arrest, leading to apoptosis in cancer cells [30,31,32,34].

**Figure 9:**
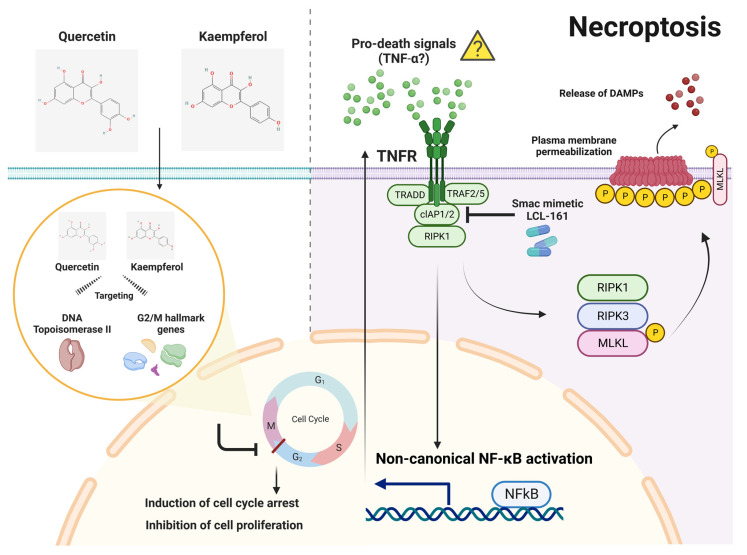
A proposed mechanism of necroptosis activation through the synergistic combination of quercetin or kaempferol with Smac mimetic LCL-161. Quercetin and kaempferol are extensively studied flavonoids with potential anticancer properties. Through network pharmacology, both quercetin and kaempferol have shown the potential to interact with multiple targets, including DNA topoisomerase II and G2/M cell-cycle hallmark genes. This interaction can result in G2/M cell-cycle arrest and inhibition of cell proliferation. In parallel, Smac mimetic LCL-161 degrades cIAP1 and cIAP2, activating a non-canonical NF-κB signaling pathway that generates pro-death signals, potentially including autocrine TNF-α. In the context of slow proliferation of CCA cells induced by quercetin or kaempferol, the accumulation of pro-death signals generated by Smac mimetic LCL-161 triggers necroptosis in the absence of cIAP1/2 and caspase activity. Necroptosis is initiated in the CCA cells expressing key components of necroptosis, including RIPK1, RIPK3, and MLKL. This process ultimately culminates in the release of damage-associated molecular patterns (DAMPs). This figure was created with BioRender.com (accessed on 15 June 2023).

In contrast to previous studies, we selected concentrations of quercetin and kaempferol that exhibit cell proliferation inhibition while inducing minimal cell death for their combination with the Smac mimetic. Additionally, in this study, we utilized the Smac mimetic LCL-161, which has entered clinical trials for both monotherapy and combination therapy in the treatment of certain cancers [17]. Interestingly, both quercetin and kaempferol at a concentration of 25 μM, which induced minimal cell death, exhibited the highest synergistic combination with the Smac mimetic LCL-161 when caspases were inhibited in two different CCA cell lines. Further investigation is required to explore the precise underlying molecular mechanisms of how flavonoids (quercetin and kaempferol) and Smac mimetics synergistically enhance sensitivity to necroptotic cell death. Previous studies in various types of cancer have shown that treatment with Smac mimetic monotherapy can induce the expression and secretion of TNF-α via a non-canonical NF-κB signaling [15,16]. Although, the concentration of Smac LCL-161 used in this study can effectively induce both cIAP1 and cIAP2 degradation (Supplementary Figure S1), it only triggered minimal cell death. Since both quercetin and kaempferol can arrest CCA cells in the G2/M phase, inhibiting cell proliferation, this may allow the accumulation of pro-death signals received from Smac mimetic treatment, possibly TNF-α, to induce CCA cell death (Figure 9). Therefore, the measurement of TNF-α secretion warrants further investigation. Additionally, the validation of the therapeutic potential of the combination treatment needs further investigation in an in vivo CCA tumor model.

To examine whether the combination treatment is specific to CCA cells, we performed the same experiment setting in a non-tumor cholangiocyte, MMNK-1, as a representative of non-tumor cells. The combination treatment did not trigger cell death when caspases were inhibited; however, treatment with quercetin or kaempferol alone significantly induced cell death, most likely apoptosis, in a dose-dependent manner. The non-tumor cholangiocyte was more sensitive to a single treatment with quercetin than kaempferol. In contrast to CCA cell lines (RMCCA-1 and HuCCT-1) which express key necroptotic proteins, including RIPK1, RIPK3, and MLKL [8], MMNK-1 does not express RIPK3 and expresses a low level of MLKL. Therefore, the combination treatment in the presence of caspase inhibitors does not switch quercetin- or kaempferol-induced apoptosis to necroptosis. In fact, the presence of caspase inhibitors almost completely inhibited quercetin- or kaempferol-induced cell death, suggesting that the single treatment of quercetin or kaempferol induces caspase-dependent cell death. Consistent with our previous studies, which demonstrated that the mRNA expression of RIPK3 and MLKL is lower in the normal bile duct compared to CCA tissues [8], we further validated these findings in tissue samples from CCA patients using immunohistochemistry (IHC). The IHC results showed a similar expression pattern for both RIPK3 and MLKL [47].

Moreover, to evaluate the therapeutic significance of the target genes of quercetin and kaempferol in the G2/M gene signature, which includes *CCNA2*, *CCNB1*, *CCNB2*, *CDC25B*, *CDC25C*, *CDK1*, *ABL1*, *AURKB*, *CHEK1*, and *PLK1*, we used this G2/M gene signature to stratify patients into two groups. Interestingly, CCA patients with high expression of this G2/M gene signature were associated with unfavorable survival outcomes (*p* = 0.017), indicating that these genes might be druggable targets for CCA therapy. Although, when analyzing the expression of each gene with patients’ survival, only *PLK1* turned out to be significantly associated with patients’ survival (*p* = 0.026) (Supplementary Figure S2). This finding indicates that the stronger association obtained from the G2/M gene signature might result from the interaction of these genes in the regulation of the G2/M cell cycle. In concordance with our study, previous studies have demonstrated a correlation between high *PLK1* expression and unfavorable prognosis in CCA patients as well as the effectiveness of inhibiting PLK1 in CCA cells [56,57,58]. While PLK1 inhibitors such as BI2536 and GW843682X have been predicted to be effective drugs for CCA patients with a poor prognosis [59], PLK1 inhibitors such as NMS-1286937 (onvansertib) and BI2536, BI6727 (volasertib) have been evaluated in clinical trials and are generally well-tolerated, and their clinical efficacy is partially responsive with a monotherapy, particularly in cancers at advanced stages [60]. These results further support the potential therapeutic use of quercetin or kaempferol in targeting multiple genes in the G2/M cell cycle.

## 5. Conclusions

This is the first study to explore the novel potential combination of flavonoids, specifically quercetin and kaempferol, with Smac mimetics. This novel combination shows promise for the development of a necroptosis-based therapeutic approach, not only for CCA but also for other cancer types.

## Data Availability

Not applicable.

## Supplementary Materials

The following supporting information can be downloaded at: https://www.mdpi.com/article/10.3390/nu15143090/s1, Figure S1: Smac mimetic LCL-161 triggers degradation of cIAP1 and cIAP2 in RMCCA-1 and HuCCT-1. RMCCA-1 and HuCCT-1 were treated with 5 μM or 25 μM Smac mimetic LCL-161, respectively for indicated time points. The expression of cIAP1 and cIAP2 was determined by Western blot analysis. β-actin served as loading control; Figure S2: The association between *PLK1* expression and survival of CCA patients. A Kaplan–Meier curve was used to compare the survival time between the high and low *PLK1* expression groups. The median was used as the cut-off to stratify patients into the high and low expression.

## Abbreviations

CCA, Cholangiocarcinoma; IAPs, inhibitors of apoptosis proteins; RIPK1 and RIPK3, receptor-interacting protein kinase 1 and 3; MLKL, mixed lineage kinase domain-like; DAMPs, damage-associated molecular patterns; cIAP1/2, cellular inhibitor of apoptosis proteins 1 and 2; Smac, second mitochondria-derived activator of caspases; Q, Quercetin; K, Kaempferol; LZ, LCL-161 + zVAD-fmk.
