# Supplementary figures and images for: Natural Flavonoids Quercetin and Kaempferol Targeting G2/M Cell Cycle-Related Genes and Synergize with Smac Mimetic LCL-161 to Induce Necroptosis in Cholangiocarcinoma Cells
Source: Nutrients. 2023 Jul 10;15(14):3090. doi: 10.3390/nu15143090 (PMC10384840; doi:10.3390/nu15143090)

A.

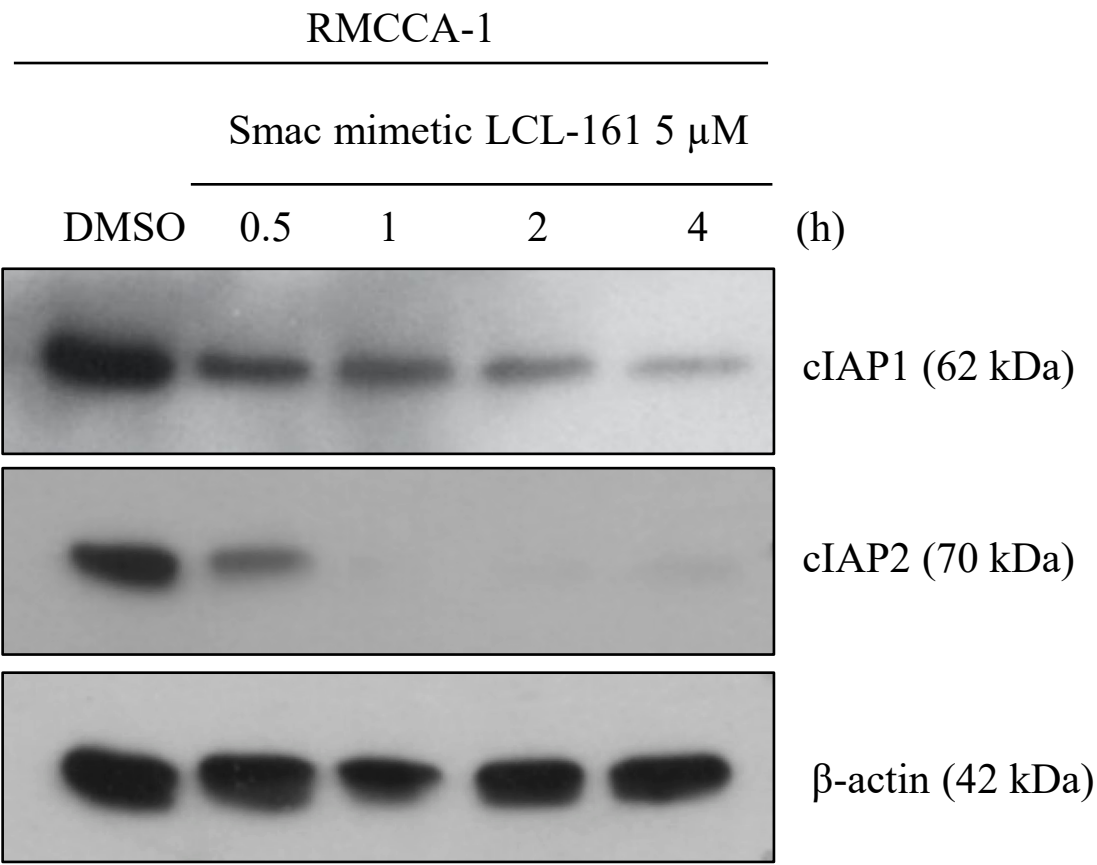

B.

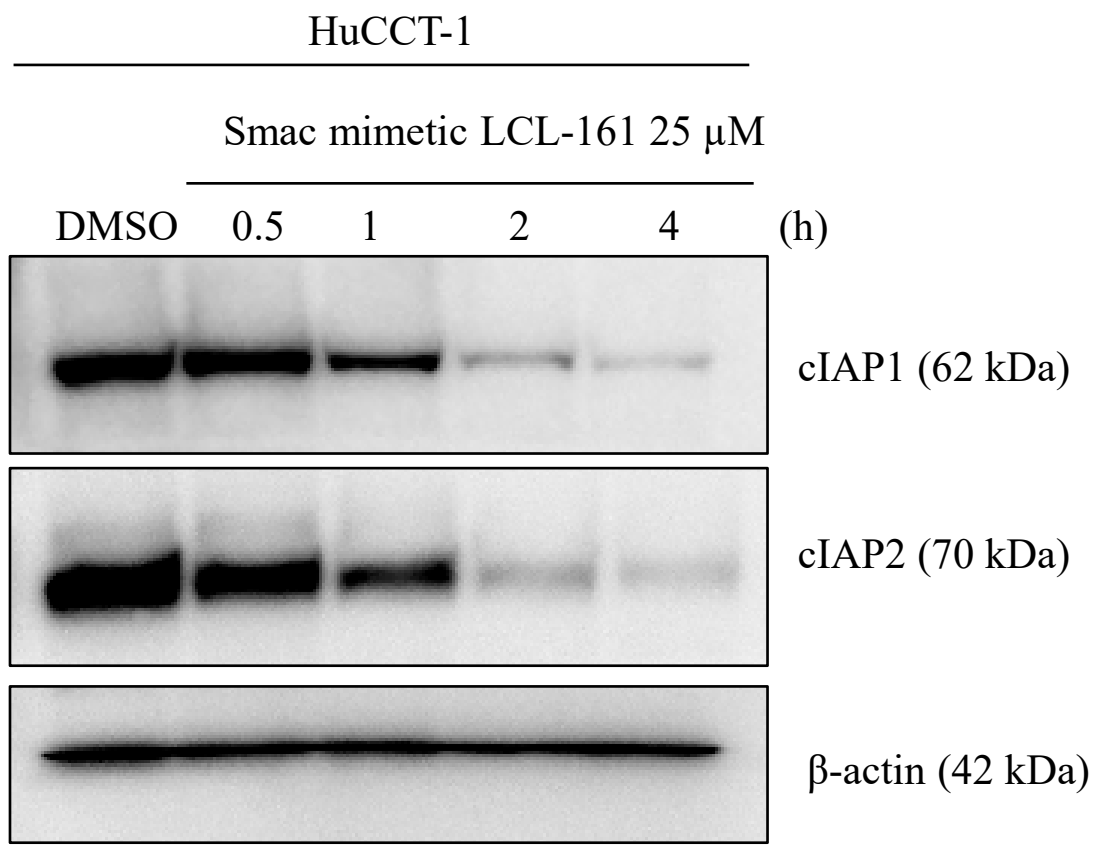

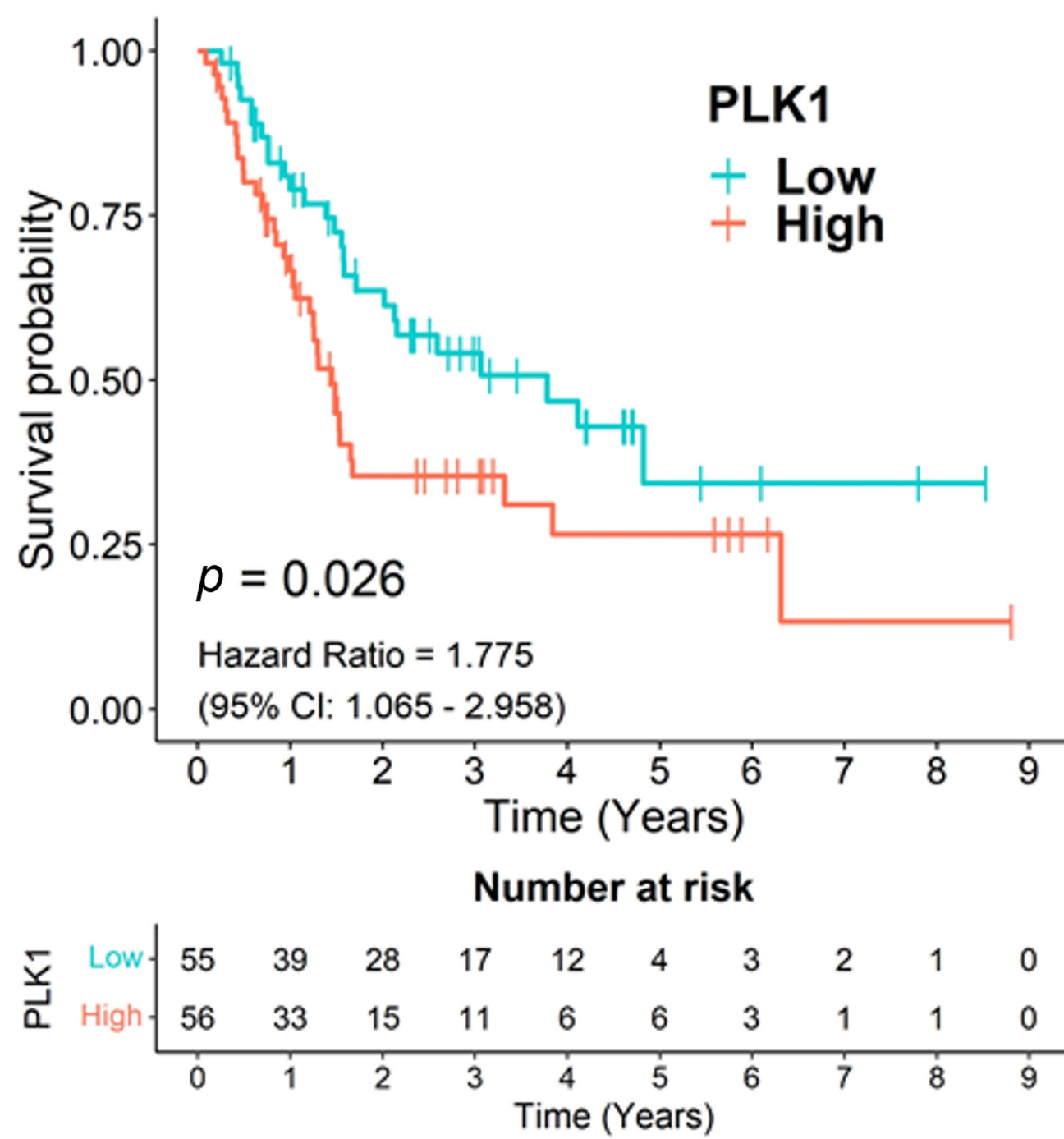

Supplement: Supplementary file 1 [file nutrients-15-03090-s001.zip › nutrients-2483980-supplementary.pdf]
